# Cognitive Contributors of Backward Walking in Persons with Multiple Sclerosis

**DOI:** 10.1155/2023/5582242

**Published:** 2023-08-11

**Authors:** Taylor N. Takla, Alexis N. Chargo, Ana M. Daugherty, Nora E. Fritz

**Affiliations:** ^1^Neuroimaging and Neurorehabilitation Laboratory, Wayne State University, Detroit, MI, USA; ^2^Translational Neuroscience Program, Wayne State University, Detroit, MI, USA; ^3^Department of Psychology, Wayne State University, Detroit, MI, USA; ^4^Institute of Gerontology, Wayne State University, Detroit, MI, USA; ^5^Department of Health Care Sciences, Wayne State University, Detroit, MI, USA; ^6^Department of Neurology, Wayne State University, Detroit, MI, USA

## Abstract

**Purpose:**

Individuals with multiple sclerosis (MS) are at an increased fall risk due to motor and cognitive dysfunction. Our past studies suggest that backward walking (BW) velocity predicts fall risk; however, specific cognitive domains associated with BW velocity remain understudied. The goal of this study was to determine the specific contributions of cognitive functioning to BW velocity in persons with MS. We hypothesized that better visuospatial memory, verbal immediate recall, and faster information processing speed would contribute to faster BW velocity, and deficits in these domains would partially account for disease severity-related impairment in BW velocity.

**Methods:**

Participants completed demographic questionnaires, walking tests, and cognitive assessments. Applied structural equation modeling was used to test our hypothesized model of competing cognitive mediators. Within the model, disease severity was a predictor of BW via three intercorrelated cognitive mediators.

**Results:**

Participants included 39 individuals with relapsing-remitting MS. Results indicated that 35.3% of the significant total effect of disease severity on BW was accounted for by specific cognitive deficits. Verbal immediate recall had the largest contribution, followed by visuospatial memory and information processing speed.

**Conclusions:**

When examining the unique effects of cognitive domains on disease severity-related deficits in BW, a meaningful source of impairment related to visuospatial memory and verbal immediate recall was demonstrated. Considering the utility of BW velocity as a predictor of falls, these results highlight the importance of assessing cognition when evaluating fall risk in MS. Cognitive-based intervention studies investigating fall prevention may find BW as a more specific and sensitive predictor of fall risk than forward walking.

## 1. Introduction

Multiple sclerosis (MS) is the most common CNS demyelinating disease. MS affects more than 700,000 individuals in the United States, and its prevalence has steadily increased over the past 5 decades [1]. MS-related pathology commonly affects the cerebellum and corpus callosum, two areas involved in motor and cognitive functioning [2–4]. Impairments in these regions contribute to two of the most common symptoms in MS: walking difficulty and cognitive dysfunction [5, 6]. As a result of motor and cognitive impairments, persons with MS (pwMS) are at an increased risk of accidental falls when compared to age-matched healthy controls [7]. Previous studies have indicated that more than 50% of pwMS fall at least once within a 3-6-month observation period [7–10], resulting in serious injury, increased fear of falling, decreased quality of life, and curtailed activity [11–13]. Therefore, preventing injurious falls in pwMS is of great importance.

Backward walking (BW) is a nonautomatic motor skill that requires greater attention, cognitive resources, and postural control [14–16] when compared to forward walking (FW). Among pwMS, BW is associated with greater walking differences and stepping deficits compared to FW [17, 18]. Previous work from our lab has shown that slower BW speed is significantly associated with greater fall risk in pwMS [19] and that BW velocity was the strongest unique predictor of falls compared to variables of stride length, double support time, age, disease severity, and symptom duration [20]. We have also shown that BW is a sensitive functional marker of fall risk regardless of the cognitive status of the pwMS [21], which supports clinical translation of the assessment.

Research examining factors contributing to falls has focused almost exclusively on balance and motor function. Though gait difficulties [22–24] and impairments in postural control [24, 25] are related to increased fall risk, motor deficits alone cannot account for the high prevalence of falls among pwMS [9, 26]. Therefore, recent studies have begun to examine the role of cognition in falls. Studies in pwMS have indicated that fallers show significantly greater deficits in attention and verbal function as compared to nonfallers [27, 28]. Furthermore, frequent fallers experience slower information processing speed as compared to single-time fallers [29]. Dual-task studies have revealed an interaction of walking and cognition in predicting fall risk in pwMS [30, 31]. One leading hypothesis is that individuals with impaired cognitive function are more prone to distraction during walking, leading to an increased risk of falls [32].

Given the compelling evidence of falls associated with cognitive deficits, identifying the specific cognitive architecture of walking ability as a functional indicator of fall risk and its vulnerability in pwMS may have important applications for clinical trial design. Studies in FW confirm the link between cognitive function and gait speed in mild cognitive impairment [33, 34] and suggest that slower FW velocity is predictive of cognitive decline in older adults [35, 36]. Because BW requires greater cognitive demand, it may be a more sensitive functional marker for the complex cognitive-motor function that is vulnerable in MS. Yet, few studies have explored the relation of cognitive functioning to BW. Our lab has shown that poorer information processing speed was associated with slower BW velocity in pwMS [37], while others have reported a correlation between poor verbal memory and slow BW velocity in people with dementia [14]. BW is also expected to rely on visuospatial memory more than FW because of greater demands on proprioception [38] due to the absence of visual input. This is consistent with impairments in visuospatial memory negatively impacting the memorization of landmarks during locomotion [39]. However, the relation of performance in specific cognitive domains to BW speed in the MS population remains relatively understudied.

Therefore, the goal of this study was to determine the specific contributions of cognitive functioning to BW velocity in pwMS. We hypothesized that better visuospatial memory, verbal immediate recall, and faster information processing speed would contribute to faster BW velocity and that worse performance in these domains would partially account for disease severity-related impairment in walking velocity.

## 2. Methods

### 2.1. Participants

In this cross-sectional study, a convenience sample of individuals with relapsing-remitting MS (RRMS) was recruited from local MS support groups and the Wayne State University MS Clinic. All study procedures were approved by the Wayne State University Institutional Review Board, and all participants provided written informed consent. Participants were included if they met the following inclusion criteria: age 18-75 years, ambulatory without physical assistance (Patient Determined Disease Steps (PDDS) ≥ 6), and stable on immunomodulatory therapy (if applicable) for 3 months prior to the study visit. Exclusion criteria included MS exacerbation within 2 months of the study visit, corticosteroid treatment in the past 30 days, acute orthopedic injuries that would interfere with walking, diagnosis of a neurological condition other than MS, and inability to follow study-related commands.

### 2.2. Measures

In a single session, participants completed demographic information including age, sex, disease severity with PDDS [40–43], and symptom duration, participated in walking tests, and completed cognitive assessments.

BW speed was captured with the 16-foot GAITRite electronic walkway (MAP/CIR, Franklin, NJ). The GAITRite is embedded with sensors that capture footfalls in real time; it is reliable and valid for use in pwMS [44]. The GAITRite calculates spatial and temporal parameters of gait, including velocity. Per the methods of our laboratory [20, 37], participants were instructed to begin walking 2 meters before the GAITRite walkway to allow for acceleration and stop 2 meters after the GAITRite to allow for deceleration, while walking at a comfortable, safe pace and looking ahead rather than at their feet. Four trials were recorded, and the data were averaged. All participants wore a gait belt and were accompanied by a member of the research team to ensure safety and minimize path deviations.

Cognitive functioning was assessed with the Brief International Cognitive Assessment for MS (BICAMS) [45], which includes tests of three cognitive domains that have been validated with clinical samples [46]. The Symbol Digit Modalities Test (SDMT) [47] assesses information processing speed—a domain of function that describes cognitive efficiency typically measured by task speed or accuracy during timed tasks. Participants were asked to determine the number belonging with each symbol presented using a key of nine number/symbol pairs. The SDMT was presented orally, and the number of correct responses in 90 seconds was used for analysis. The SDMT is a reliable and valid instrument for examining information processing speed [48], including in pwMS [49]. A second domain of function assessed by the Brief Visuospatial Memory Test-Revised (BVMT-R) [50] was visuospatial memory—the ability to recall an object's relative location in space. Participants were asked to view six shapes organized in a 2 × 3 matrix for 10 seconds, after which they were asked to draw as many shapes as accurately as possible. Immediate recall was assessed as the total number of correct responses across three trials, which has support for strong construct validity [51]. The standardized administration of the task included a delayed free recall of the shapes after a 25-minute delay, which was not included in the reported hypothesis tests. Finally, the California Verbal Learning Test (CVLT) [52] was used to assess auditory-verbal learning and immediate recall—or the ability to acquire, manipulate, and retain verbal information with rehearsal. Participants listened to a list of 16 words read aloud by the examiner before freely recalling the list. The list was repeated for a total of five trials, each followed by free recall. The total number of items correctly recalled across the five trials was an index of verbal immediate recall and was calculated as a *z*-score, which has shown good construct validity in pwMS [53]. Though traditionally CVLT is referred to as an index of verbal learning, the participant is required to maintain, update, and manipulate the list of words using order or semantic classes, all of which are functions of working memory that support immediate recall [54]. As we are reporting a summary index of correct recall across trials, we believe that this reflects the underlying working memory functions generally across the 5 trials of immediate recall rather than verbal learning. After immediate recall, standardized administration of the task includes a second “distractor” list with single trial recall followed by recall of the first list (a measure of memory interference) and delayed recall of the first list after 25 minutes. The interference cost and delayed recall scores were not included in the reported hypothesis tests. Normalized scores account for potential bias in the assessment related to years of education [55], which were used in subsequent hypothesis testing.

### 2.3. Statistical Analyses

Primary hypotheses of cognition mediating the effects of PDDS on walking velocity were tested in Mplus [56] (v 7.4), and initial data screening and correlation analyses were generated in SPSS (v. 28; IBM, Armonk NY). Prior to hypothesis testing, all univariate, continuous scale distributions were screened and found to be approximately normal (all skew z < |1.53|) and without univariate outliers (all z < |2.3|), nor was there indication of multivariate outliers (Mahalanobis distance, critical *χ*^2^ = 18.47, *α* = 0.001).

Hypothesis tests were specified in applied structural equation modeling. The hypothesized model of competing cognitive mediators was specified with path modeling as PDDS predicting BW via three intervening cognitive mediators (CVLT, BVMT, and SDMT) that were allowed to intercorrelate. As a control comparison, a similar model tested the effects of PDDS on FW via the same cognitive mediators. The mediators were expected to correlate due to some overlapping cognitive processes; therefore, an alternative model that could account for a similar pattern of correlations would be a general cognitive deficit across tasks. We tested this alternative model by including a latent general cognition construct (GCog) that was reflective of the three cognitive tasks, for which CVLT was set with a loading of 1 for local identification, and the loadings of the other indicators were freely estimated. Age (continuous) was included as a covariate of walking velocity and cognitive performance in all models. All path coefficients are reported as unstandardized estimates, which can be interpreted on the original variable scales. Model fit for each model was assessed by a compendium of indices [57]: chi-square (*χ*^2^) nonsignificance (*α* = 0.05), comparative fit index (CFI) exceeding 0.90, root mean square error of approximation (RMSEA) below 0.08, and standardized root mean residual (SRMR) below 0.10 collectively indicated excellent model fit to the observed data. Mediation was tested as indirect effects [58] with bias-corrected bootstrapped 95% confidence intervals (BS 95% CI; 5000 draws) not including zero. Because statistical power is differential across portions of the model testing mediation, interpretation of BS 95% CI is the recommended best practice [59], and we provided secondary description of Sobel's *z*-tests with significance testing. In addition to reporting the total, direct, and indirect effects of PDDS on walking velocities, the mediated effect magnitudes were described by percentage of cumulative absolute effect.

## 3. Results

A clinical sample of 39 (89.7% female) participants with RRMS was recruited from the Metro Detroit area to participate in this study. Participants (age 36–69 years) had an average symptom duration of 17.26 years (SD = 11.80). See Table 1 for a complete demographic profile of the sample.

To examine the relation between PDDS, BW velocity, and each hypothesized cognitive mediator, Pearson's *r* correlations were computed (Table 2). A negative correlation between PDDS and CVLT (*r* = −0.428, *p* < 0.01), BVMT (*r* = −0.450, *p* < 0.01), and SDMT (*r* = −0.386, *p* < 0.05) indicates that greater disease severity was associated with decreased verbal immediate recall, visuospatial working memory, and slowed information processing speed, respectively. A general positive correlation between BVMT and BW velocity (*r* = 0.345, *p* < 0.05) indicates that greater cognitive performance was associated with faster BW, with a similar (not significant) trend noted for CVLT (*r* = 0.108, *p* = 0.53) and SDMT (*r* = 0.283, *p* = 0.09).

### 3.1. Competing Cognitive Mediators of the Effect of PDDS on Backward Walking Velocity

**Figure 1:**
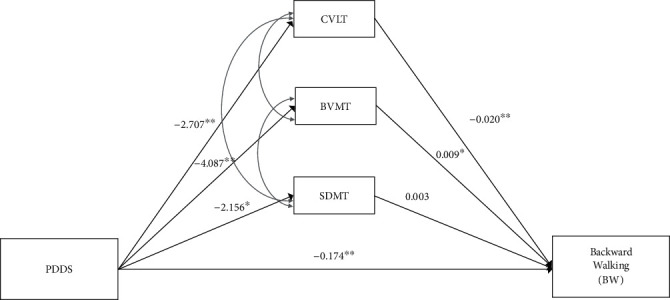
A path diagram of the competing mediation model. Depicted is the outcome BW that is predicted by PDDS, via three cognitive tasks: CVLT, BVMT, and SDMT. In the model diagram, straight arrows represent regression paths, with the unstandardized path coefficient (*b* weights) reported. All cognitive measures were allowed to intercorrelate, represented by the curved double-headed arrows. ^∗^*p* < 0.05 and ^∗∗^*p* < 0.01. Age was a covariate in analysis that is not illustrated.

The competing mediation model (see Figure 1) tested performance on multiple cognitive tasks as mediators of PDDS on BW and was deemed to have excellent fit as indicated by a nonsignificant chi-square (*χ*^2^ = 3.04, df = 3, *p* = 0.39), in addition to other acceptable fit indices (RMSEA = 0.02, CFI = 0.99, SRMR = 0.05). The model accounted for 57.5% of the total variability in BW (*R*^2^ = 0.575, *p* < 0.001).

There was a significant total effect of PDDS on BW (*b* = −0.163, *p* < 0.001). Evidence of mediation was indicated by a significant cumulative indirect effect with a BS 95% CI not overlapping zero. 35.3% of the indirect effect of PDDS on BW was attributed to specific cognitive deficits often associated with MS. As indicated by BS 95% CI not overlapping zero, CVLT (indirect = 0.05, BS 95% CI: 0.01, 0.12; *z* = 2.18, *p* = 0.03) and BVMT (indirect = −0.04, BS 95% CI: -0.10, -0.01; *z* = −1.79, *p* = 0.07) mediated the effect of PDDS on BW, and the small mediated effect via SDMT was not supported (indirect = −0.006, BS 95% CI: -0.04, 0.01; *z* = −0.52, *p* = 0.61). Considering potential unique contributions of each of the cognitive mediators, CVLT had the largest unique contribution, followed by BVMT and SDMT, respectively (see Table 3). These results suggest a plausible cognitive architecture of MS disease effects on BW with relatively greater contributions of verbal immediate recall and visuospatial memory, whereas slowed information processing speed was associated with slowed BW but did not mediate the effects of disease severity.

### 3.2. Testing General Cognitive Deficit as an Alternative Model Account of Mediated Effects

**Figure 2:**
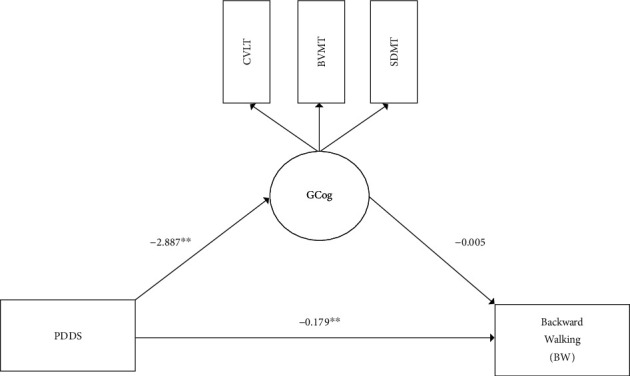
A path diagram of the general cognition (GCog) mediation model. Depicted is the outcome BW that is predicted by PDDS, mediated by the latent GCog. The arrows depicted represent regression paths, with the unstandardized path coefficients (*b* weights) reported. ^∗^*p* < 0.05 and ^∗∗^*p* < 0.01.

An alternative account of the effects observed would be a general cognitive deficit opposed to differential effects across cognitive domains, as there are shared cognitive processes across CVLT, BVMT, and SDMT task performance. This general cognitive deficit was tested in an alternative model where general cognition (GCog)—reflective of CVLT (loading fixed = 1.0), BVMT (loading = 1.53, *p* < 0.001), and SDMT (loading = 0.70, *p* < 0.001)—was substituted as the mediator between PDDS and BW (see Figure 2). This alternative model, however, did not have as good of fit to the data (*χ*^2^ = 17.35, df = 8, *p* < 0.05, RMSEA = 0.17, CFI = 0.88, SRMR = 0.09). Further, the model that included a general cognitive construct accounted for only 47.0% of the total variability in BW (*R*^2^ = 0.470, *p* < 0.001), whereas the model including distinct cognitive domains accounted for 57.5% of the total variability in BW (*R*^2^ = 0.575, *p* < 0.001). Therefore, the model using a general cognitive construct explains less total variance as compared to including separate cognitive domains. Further, the percentage of the PDDS effect on BW that was mediated by cognition was less when modeled as a general deficit (7.0%) as compared to when modeling task-specific mediators (totaling 35.32%). There was no evidence of an indirect effect of PDDS on BW via general cognition (indirect = 0.02, *z* = 0.61, *p* = 0.54; BS 95% CI: -0.03, 0.09) providing little evidence in support of a general cognitive deficit accounting for the relation between PDDS and BW.

### 3.3. Competing Cognitive Mediators of PDDS on Forward Walking Velocity

Repeating the analysis of competing mediators to instead predict FW, we find a smaller contribution of cognition to performance: CVLT mediated the effect of PDDS on FW (indirect = 0.049, BS 95% CI: 0.01, 012; *z* = 2.20, *p* = 0.028), but BVMT (indirect = 0.001, BS 95% CI: -0.03, 0.03; *z* = 0.08, *p* = 0.93) and SDMT (indirect = −0.014, BS 95% CI: -0.06, 0.01; *z* = −1.20, *p* = 0.23) did not. Collectively, cognitive correlates accounted for less of the effect of PDDS on FW (15.84% cumulative) as compared to what we found when predicting BW (see Table 3). The model in total accounted for 83% of variance in FW (*R*^2^ = 0.834, *p* < 0.001) and had moderately good fit to the observed data (*χ*^2^ = 9.34, df = 3, *p* = 0.03, RMSEA = 0.23, CFI = 0.95, SRMR = 0.04).

Taken together, PDDS was associated with slowed BW in part due to deficits in intervening visuospatial memory and verbal immediate recall function, and in largest part verbal immediate recall as measured by CVLT. Although differences in verbal immediate recall also partially accounted for PDDS effects on FW, the proportion of the effect related to cognition was smaller than that in BW and did not suggest visuospatial memory and information processing speed as contributing to disease-related deficits in FW.

## 4. Discussion

Previous work from our lab has demonstrated that BW velocity can be used as a sensitive predictor of fall risk in pwMS [19, 20], as it is a novel motor skill that requires greater cognitive demand than FW [14, 16]. While previous studies have acknowledged the importance of BW as an indicator of fall risk in pwMS, there has been little consideration of the cognitive architecture that supports BW. Cognitive correlates to walking velocity have been reported before [33, 34, 60], but few have tested specific domains of cognition to account for disease severity-related deficits in BW velocity.

We address this limitation in the current study by examining the unique contributions of visuospatial memory, information processing speed, and verbal immediate recall on BW velocity in pwMS. In general, our hypothesis that better performance in these cognitive domains correlates with faster BW velocity was supported. Disease severity measured by PDDS significantly predicted BW velocity, and 35.3% of this effect was accounted for by lower ability scores in specific cognitive domains. CVLT and BVMT performance—indicators of verbal immediate recall and visuospatial working memory—mediated the effect of disease severity (PDDS) on BW. Performance on SDMT was independently associated with slowed BW velocity, but it did not mediate the effects of PDDS on BW. When considering the unique contributions on BW velocity from each cognitive assessment, CVLT had the greatest contribution in mediating the relation of PDDS on BW velocity, followed by BVMT, then a negligible effect of SDMT.

CVLT, BVMT, and SDMT measure verbal immediate recall, visuospatial working memory, and information processing speed, respectively. Due to the reliance on proprioception and memory of the environment to BW, we had anticipated that visuospatial memory (BVMT) would be the strongest mediator, but this was not the case. CVLT immediate recall was determined as the strongest unique contributor in mediating the effects of PDDS on BW. This is consistent with the previous reports of worse performance on the CVLT correlated with slower FW velocity in pwMS [27]; here, we expand this finding to show a unique effect above and beyond the other cognitive domain effects on BW. Low CVLT scores are also predictive of greater fall risk in pwMS [27], and based on this study, BW may be a sensitive functional marker in the clinic to detect this source of risk.

Indeed, we found that BW was more sensitive than FW to the PDDS-related effects on cognition. Given the increase in cognitive demand during BW, individuals may be recruiting other brain regions than they typically would during FW. It has been shown that BW and backward stepping is associated with greater levels of cortical activation than their forward counterpart, specifically showing enhanced prefrontal and parietal cortex activation [61, 62], the same regions involved in verbal immediate recall measured by the CVLT [63]. Furthermore, research has also demonstrated a role for the cerebellum in working memory [64, 65], a brain region also critical for motor skill execution of bilateral tasks such as BW. Taken together, these findings support the notion that BW recruits brain regions associated with verbal memory, including working memory function that supports immediate recall.

The BVMT, a visuospatial memory index, also mediated the effect of disease severity on BW, albeit having a smaller unique contribution than CVLT. Visuospatial memory is likely to be important during BW due to the lack of visual input as participants walk backward while facing forward. In the absence of visual cues in the direction in which the participant is moving, the visuospatial memory system may be heavily relied upon. The results of this study support a role for visuospatial memory in BW but call attention to the importance of verbal immediate recall.

Prior findings in the MS literature have highlighted overlapping representation of cognitive domains by performance on the CVLT and BVMT. While BVMT examines visuospatial episodic memory, the encoding and immediate recall of episodic information is partially dependent on working memory [66]. In part due to this overlap in cognitive processes [67], the source of performance deficits on the CVLT immediate recall is suggested to be similar to that on the BVMT [51, 68, 69]. Coinciding evidence of similar activation patterns in brain regions, including the prefrontal cortex and hippocampus, when engaged in visuospatial and immediate recall tasks [70] further suggests some lack of specificity in the assessments. These known limitations of the cognitive assessments motivated testing an alternative model of general cognition mediating the effects of PDDS on BW. Critically, we found little support for this model: it did not reliably fit the observed data, and it explained a fraction of the variance in BW. This alternative model comparison provides additional support for interpreting the unique and specific cognitive tasks as separate but correlated.

Interestingly, deficits in information processing speed measured by the SDMT did not mediate the effects of disease severity on BW velocity. This finding was unexpected considering that previous data from our lab has shown that slower information processing speed was correlated with slower BW velocity in pwMS [37]. In this sample, we observed moderate correlations of SDMT with walking velocity; however, in the competing mediator model, the unique effect of SDMT above the contribution of CVLT and BVMT was small and did not account for PDDS-related effects on BW. Previous work in pwMS has demonstrated that the SDMT was shown to have components from multiple cognitive domains, including working memory and visuospatial memory [71, 72], suggesting that SDMT performance may be better interpreted as a measure of general cognitive abilities rather than a specific measure of information processing speed [73]. Therefore, impairments in processing speed could be indicative of deficits pertaining to general disease-related changes in cognition, which may explain why the SDMT had a negligible unique effect mediating disease severity (PDDS) on BW. Taken together, these findings demonstrate that by considering the specific cognitive domains of verbal immediate recall, visuospatial memory, and information processing speed, we can strengthen the understanding of how disease severity relates to BW velocity.

In clinical trials that are targeting reduction of fall risk with cognitive intervention, our findings support the use of BW velocity as a primary assessment. Prospective fall data as a primary outcome measure may be problematic due to the unknowable frequency of previous falls and the possibility of having no fall occurrence within the clinical trial period for at least some participants, which may not truly reflect reduction in fall risk. To supplement prospective fall data, BW can be used as a feasible clinical measure that can be easily assessed in everyone on repeated occasions and is a continuous scale that offers good reliability and power for analysis. Further, BW has been validated to be predictive of fall risk, regardless of cognitive status, and the current study has shown it to be sensitive to the cognitive vulnerabilities in MS, with greater sensitivity than FW.

### 4.1. Limitations

The results from this study should be considered in terms of strengths and limitations. Our sample consisted of primarily female participants with RRMS, with only few males included. However, considering that MS affects women more than men at a 3 : 1 ratio [74], the sample is representative of the clinical population. Importantly, we recognize that our sample is relatively low disability, with an average PDDS score of 3.26 (Table 1), limiting the ability to generalize our findings to the broader MS community. Additionally, we acknowledge that our sample size is relatively small. Future studies should examine the cognitive correlates of backward walking in a larger sample size with greater variability of disease severity. Further, the findings highlighted were from a single time point of assessment; therefore, we did not examine the potential longitudinal effects of cognition and how this might differentially affect BW abilities in pwMS. Importantly, the findings presented here highlight the effects of only three cognitive domains: verbal immediate recall, visuospatial memory, and information processing speed. Previous work has demonstrated that performance in other cognitive domains such as attention and sensory-motor functions including vision and proprioception, can be predictive of fall risk in healthy older adults [32, 75]. In pwMS, deficits in attention have also been associated with an increased fall risk [76], and visuo-proprioceptive training has been shown to reduce fall risk and increase FW velocity [77]. Impairments in BW relating to fall risk may be due to declines in these specific domains that make it more challenging for individuals to walk while simultaneously engaging in other high-demand motor and cognitive functions. Moreover, greater dual-task cost is associated with executive dysfunction and decreased walking velocity in pwMS [31, 60]; therefore, future studies should consider the impact of other domains of cognition on mediating BW velocity.

## 5. Conclusion

To our knowledge, the findings demonstrated from this study are the first to examine the distinct, but correlated, cognitive domains to improve the description of PDDS-related deficits in BW for pwMS. When we compared the unique effects across cognitive assessments, meaningful sources of impairment related to visuospatial memory and verbal immediate recall were identified. Our results provide insight into potential neural and cognitive targets to prevent falls in pwMS. Given these findings and considering the clinical advantages of BW [20] over FW, clinical trials that are aimed at reducing the prevalence of falls in pwMS using cognitive interventions should consider incorporating BW as a specific functional indicator of fall risk.

## Figures and Tables

**Table 1:** Sample description.

Variable	Descriptive statistic
Sample size	39
Female, *n* (%)	35 (89.7%)
Age (years)	50.87 ± 9.48
Education (years)	16.82 ± 2.26
Right-handed, *n* (%)	34 (87.2%)
Symptom duration (years)	17.26 ± 11.80
Disease severity (PDDS)	3.26 ± 2.05
Assistive device use, *n* (%)	21 (53.8%)
Disease-modifying therapy, *n* (%)	37 (94.9%)

Note: demographic profile of clinical RRMS sample recruited from Metro Detroit. Sample means and standard deviations are reported (*M* ± SD). PDDS: Patient Determined Disease Steps.

**Table 2:** Bivariate correlations among study variables.

Variable	1	2	3	4	5	6	7
1. Age	1						
2. PDDS	0.187	1					
3. CVLT	0.069	-0.428^∗∗^	1				
4. BVMT	-0.192	-0.450^∗∗^	0.722^∗∗^	1			
5. SDMT	-0.184	-0.386^∗^	0.518^∗∗^	0.533^∗∗^	1		
6. Backward walking (BW)	-0.174	-0.677^∗∗^	0.108	0.345^∗^	0.283	1	
7. Forward walking (FW)	-0.058	-0.869^∗∗^	0.152	0.235	0.322	0.780^∗∗^	1

Note: Pearson's *r* correlations describing the bivariate relation between age, PDDS, hypothesized cognitive mediators, and walking velocity. ^∗^*p* < 0.05 and ^∗∗^*p* < 0.01 unadjusted.

**Table 3:** Effect composition of competing cognitive mediator and comparison control models.

Effect composition	BW	FW
Total effect	-0.163, *p* < 0.001	-0.304, *p* < 0.001
% direct effect	64.68	84.16
% cumulative indirect effect	35.32	15.84
CVLT	19.70	12.13
BVMT	13.38	0.25
SDMT	2.23	3.47

## Data Availability

Materials and data for this study are available upon request.

## Abbreviations

MS: Multiple sclerosis
pwMS: Persons with multiple sclerosis
BW: Backward walking
FW: Forward walking
PDDS: Patient Determined Disease Steps
RRMS: Relapsing-remitting multiple sclerosis
BICAMS: Brief International Cognitive Assessment for Multiple Sclerosis
SDMT: Symbol Digit Modalities Test
BVMT-R: Brief Visuospatial Memory Test-Revised
CVLT: California Verbal Learning Test.

## Additional Points

*Preregistration*. No aspect of this study was preregistered.

## References

[1] Wallin M. T., Culpepper W. J., Campbell J. D. (2019). The prevalence of MS in the United States: a population-based estimate using health claims data. *Neurology*.

[2] Du X. F., Liu J., Hua Q. F., Wu Y. J. (2019). Relapsing-remitting multiple sclerosis is associated with regional brain activity deficits in motor- and cognitive-related brain areas. *Frontiers in Neurology*.

[3] Fritz N. E., Edwards E. M., Ye C. (2022). Cerebellar contributions to motor and cognitive control in multiple sclerosis^✰✰✰^. *Archives of Physical Medicine and Rehabilitation*.

[4] Pokryszko-Dragan A., Banaszek A., Nowakowska-Kotas M. (2018). Diffusion tensor imaging findings in the multiple sclerosis patients and their relationships to various aspects of disability. *Journal of the Neurological Sciences*.

[5] Kelleher K. J., Spence W., Solomonidis S., Apatsidis D. (2010). The characterisation of gait patterns of people with multiple sclerosis. *Disability and Rehabilitation*.

[6] Middleton L. S., Denney D. R., Lynch S. G., Parmenter B. (2006). The relationship between perceived and objective cognitive functioning in multiple sclerosis. *Archives of Clinical Neuropsychology*.

[7] Mazumder R., Murchison C., Bourdette D., Cameron M. (2014). Falls in people with multiple sclerosis compared with falls in healthy controls. *PLoS One*.

[8] Fritz N. E., Eloyan A., Baynes M., Newsome S. D., Calabresi P. A., Zackowski K. M. (2018). Distinguishing among multiple sclerosis fallers, near-fallers and non-fallers. *Multiple Sclerosis and Related Disorders*.

[9] Matsuda P. N., Shumway-Cook A., Bamer A. M., Johnson S. L., Amtmann D., Kraft G. H. (2011). Falls in multiple sclerosis. *PM and R*.

[10] Nilsagard Y., Gunn H., Freeman J. (2015). Falls in people with MS - an individual data meta-analysis from studies from Australia, Sweden, United Kingdom and the United States. *Multiple Sclerosis Journal*.

[11] Gunn H., Creanor S., Haas B., Marsden J., Freeman J. (2014). Frequency, characteristics, and consequences of falls in multiple sclerosis: findings from a cohort study. *Archives of Physical Medicine and Rehabilitation*.

[12] Peterson E. W., Cho C. C., Finlayson M. L. (2007). Fear of falling and associated activity curtailment among middle aged and older adults with multiple sclerosis. *Multiple Sclerosis*.

[13] Vister E., Tijsma M. E., Hoang P. D., Lord S. R. (2017). Fatigue, physical activity, quality of life, and fall risk in people with multiple sclerosis. *International Journal of MS Care*.

[14] Johansson H., Lundin-Olsson L., Littbrand H., Gustafson Y., Rosendahl E., Toots A. (2017). Cognitive function and walking velocity in people with dementia; a comparison of backward and forward walking. *Gait and Posture*.

[15] Thomas K. S., Hammond M., Magal M. (2018). Graded forward and backward walking at a matched intensity on cardiorespiratory responses and postural control. *Gait and Posture*.

[16] Tseng I. J., Jeng C., Yuan R. Y. (2012). Comparisons of forward and backward gait between poorer and better attention capabilities in early Parkinson's disease. *Gait and Posture*.

[17] Peterson D. S., Huisinga J. M., Spain R. I., Horak F. B. (2016). Characterization of compensatory stepping in people with multiple sclerosis. *Archives of Physical Medicine and Rehabilitation*.

[18] Wajda D. A., Sandroff B. M., Pula J. H., Motl R. W., Sosnoff J. J. (2013). Effects of walking direction and cognitive challenges on gait in persons with multiple sclerosis. *Multiple Sclerosis International*.

[19] Edwards E. M., Kegelmeyer D. A., Kloos A. D. (2020). Backward walking and dual-task assessment improve identification of gait impairments and fall risk in individuals with MS. *Multiple Sclerosis International*.

[20] Edwards E. M., Daugherty A. M., Nitta M., Atalla M., Fritz N. E. (2020). Backward walking sensitively detects fallers in persons with multiple sclerosis. *Multiple Sclerosis and Related Disorders*.

[21] Edwards E. M., Daugherty A. M., Fritz N. E. (2022). Examining the influence of cognition on the relationship between backward walking and falls in persons with multiple sclerosis. *International Journal of MS Care*.

[22] Kasser S. L., Jacobs J. V., Foley J. T., Cardinal B. J., Maddalozzo G. F. (2011). A prospective evaluation of balance, gait, and strength to predict falling in women with multiple sclerosis. *Archives of Physical Medicine and Rehabilitation*.

[23] Nilsagård Y., Denison E., Gunnarsson L. G., Boström K. (2009). Factors perceived as being related to accidental falls by persons with multiple sclerosis. *Disability and Rehabilitation*.

[24] Sosnoff J. J., Socie M. J., Boes M. K. (2011). Mobility, balance and falls in persons with multiple sclerosis. *PLoS One*.

[25] Kalron A., Achiron A. (2013). Postural control, falls and fear of falling in people with multiple sclerosis without mobility aids. *Journal of the Neurological Sciences*.

[26] Gunn H. J., Newell P., Haas B., Marsden J. F., Freeman J. A. (2013). Identification of risk factors for falls in multiple sclerosis: a systematic review and meta-analysis. *Physical Therapy*.

[27] D’Orio V. L., Foley F. W., Armentano F., Picone M. A., Kim S., Holtzer R. (2012). Cognitive and motor functioning in patients with multiple sclerosis: neuropsychological predictors of walking speed and falls. *Journal of the Neurological Sciences*.

[28] Kalron A. (2014). The relationship between specific cognitive domains, fear of falling, and falls in people with multiple sclerosis. *BioMed Research International*.

[29] Sosnoff J. J., Balantrapu S., Pilutti L. A., Sandroff B. M., Morrison S., Motl R. W. (2013). Cognitive processing speed is related to fall frequency in older adults with multiple sclerosis. *Archives of Physical Medicine and Rehabilitation*.

[30] Nilsagård Y., Lundholm C., Denison E., Gunnarsson L. G. (2009). Predicting accidental falls in people with multiple sclerosis - a longitudinal study. *Clinical Rehabilitation*.

[31] Wajda D. A., Motl R. W., Sosnoff J. J. (2013). Dual task cost of walking is related to fall risk in persons with multiple sclerosis. *Journal of the Neurological Sciences*.

[32] Mirelman A., Herman T., Brozgol M. (2012). Executive function and falls in older adults: new findings from a five-year prospective study link fall risk to cognition. *PLoS One*.

[33] Doi T., Shimada H., Makizako H. (2014). Cognitive function and gait speed under normal and dual-task walking among older adults with mild cognitive impairment. *BMC Neurology*.

[34] Knapstad M. K., Steihaug O. M., Aaslund M. K. (2019). Reduced walking speed in subjective and mild cognitive impairment: a cross-sectional study. *Journal of Geriatric Physical Therapy*.

[35] Mielke M. M., Roberts R. O., Savica R. (2013). Assessing the temporal relationship between cognition and gait: slow gait predicts cognitive decline in the mayo clinic study of aging. *Journals of Gerontology - Series A Biological Sciences and Medical Sciences*.

[36] Soumaré A., Tavernier B., Alpérovitch A., Tzourio C., Elbaz A. (2009). A cross-sectional and longitudinal study of the relationship between walking speed and cognitive function in community-dwelling elderly people. *Journals of Gerontology - Series A Biological Sciences and Medical Sciences*.

[37] Saymuah S., Laird H., Nitta M., Atalla M., Fritz N. E. (2019). Motor, cognitive, and behavioral performance in middle-aged and older adults with multiple sclerosis. *Topics in Geriatric Rehabilitation*.

[38] Thomas M. A., Fast A. (2000). One step forward and two steps back. *American Journal of Physical Medicine and Rehabilitation*.

[39] Deyzac E., Logie R. H., Denis M. (2006). Visuospatial working memory and the processing of spatial descriptions. *British Journal of Psychology*.

[40] Hohol M. J., Orav E. J., Weiner H. L. (1995). Disease steps in multiple sclerosis: a simple approach to evaluate disease progression. *Neurology*.

[41] Hohol M. J., Orav E. J., Weiner H. L. (1999). Disease steps in multiple sclerosis: a longitudinal study comparing disease steps and EDSS to evaluate disease progression. *Multiple Sclerosis*.

[42] Learmonth Y. C., Motl R. W., Sandroff B. M., Pula J. H., Cadavid D. (2013). Validation of patient determined disease steps (PDDS) scale scores in persons with multiple sclerosis. *BMC Neurology*.

[43] Marrie R. A., Goldman M. (2007). Validity of performance scales for disability assessment in multiple sclerosis. *Multiple Sclerosis*.

[44] Givon U., Zeilig G., Achiron A. (2009). Gait analysis in multiple sclerosis: characterization of temporal-spatial parameters using GAITRite functional ambulation system. *Gait and Posture*.

[45] Langdon D. W., Amato M. P., Boringa J. (2012). Recommendations for a Brief International Cognitive Assessment for Multiple Sclerosis (BICAMS). *In Multiple Sclerosis Journal*.

[46] Corfield F., Langdon D. (2018). A systematic review and meta-analysis of the Brief Cognitive Assessment for Multiple Sclerosis (BICAMS). *In Neurology and Therapy*.

[47] Sheridan L. K., Fitzgerald H. E., Adams K. M. (2006). Normative symbol digit modalities test performance in a community-based sample. *Archives of Clinical Neuropsychology*.

[48] Strober L. B., Bruce J. M., Arnett P. A. (2020). A new look at an old test: normative data of the symbol digit modalities test –oral version. *Multiple Sclerosis and Related Disorders*.

[49] Sonder J. M., Burggraaff J., Knol D. L., Polman C. H., Uitdehaag B. M. J. (2014). Comparing long-term results of PASAT and SDMT scores in relation to neuropsychological testing in multiple sclerosis. *Multiple Sclerosis Journal*.

[50] Benedict R. H. B. (1997). *Brief Visuospatial Memory Test - Revised professional manual*.

[51] Kane K. D., Yochim B. P. (2014). Construct validity and extended normative data for older adults for the Brief Visuospatial Memory Test, Revised. *American Journal of Alzheimer’s Disease and Other Dementias*.

[52] Delis D. C., Kramer J. H., Kaplan E., Ober B. A. (2000). *California Verbal Learning Test – second edition*.

[53] Stegen S., Stepanov I., Cookfair D. (2010). Validity of the California verbal learning test-II in multiple sclerosis. *The Clinical Neuropsychologist*.

[54] Nyberg L., Eriksson J. (2016). Working memory: maintenance, updating, and the realization of intentions. *Cold Spring Harbor Perspectives in Biology*.

[55] Walker L. A. S., Marino D., Berard J. A., Feinstein A., Morrow S. A., Cousineau D. (2017). Canadian normative data for minimal assessment of cognitive function in multiple sclerosis. *Canadian Journal of Neurological Sciences*.

[56] Muthén L., Muthén B. (2012). *Mplus Version 7 User’s Guide*.

[57] Raykov T., Marcoulides G. A. (2006). *A First Course in Structural Equation Modeling*.

[58] James L. R., Brett J. M. (1984). Mediators, moderators, and tests for mediation. *Journal of Applied Psychology*.

[59] Hayes A. F., Scharkow M. (2013). The relative trustworthiness of inferential tests of the indirect effect in statistical mediation analysis: does method really matter?. *Psychological Science*.

[60] Etemadi Y. (2017). Dual task cost of cognition is related to fall risk in patients with multiple sclerosis: a prospective study. *Clinical Rehabilitation*.

[61] Berchicci M., Russo Y., Bianco V. (2020). Stepping forward, stepping backward: a movement-related cortical potential study unveils distinctive brain activities. *Behavioural Brain Research*.

[62] Kurz M. J., Wilson T. W., Arpin D. J. (2012). Stride-time variability and sensorimotor cortical activation during walking. *NeuroImage*.

[63] Nowrangi M. A., Lyketsos C., Rao V., Munro C. A. (2014). Systematic review of neuroimaging correlates of executive functioning: converging evidence from different clinical populations. *Journal of Neuropsychiatry and Clinical Neurosciences*.

[64] Gottwald B., Wilde B., Mihajlovic Z., Mehdorn H. M. (2004). Evidence for distinct cognitive deficits after focal cerebellar lesions. *Journal of Neurology, Neurosurgery and Psychiatry*.

[65] Ziemus B., Baumann O., Luerding R. (2007). Impaired working-memory after cerebellar infarcts paralleled by changes in BOLD signal of a cortico-cerebellar circuit. *Neuropsychologia*.

[66] Tam J. W., Schmitter-Edgecombe M. (2013). The role of processing speed in the Brief Visuospatial Memory Test - Revised. *The Clinical Neuropsychologist*.

[67] Migliore S., Ghazaryan A., Simonelli I. (2017). Cognitive impairment in relapsing-remitting multiple sclerosis patients with very mild clinical disability. *Behavioural Neurology*.

[68] de Caneda M. A. G., Cuervo D. L. M., Marinho N. E., de Vecino M. C. A. (2018). The reliability of the Brief Visuospatial Memory Test – Revised in Brazilian multiple sclerosis patients. *Dementia e Neuropsychologia*.

[69] Sandi D., Rudisch T., Füvesi J. (2015). The Hungarian validation of the Brief International Cognitive Assessment for Multiple Sclerosis (BICAMS) battery and the correlation of cognitive impairment with fatigue and quality of life. *Multiple Sclerosis and Related Disorders*.

[70] Melrose R. J., Zahniser E., Wilkins S. S. (2020). Prefrontal working memory activity predicts episodic memory performance: a neuroimaging study. *Behavioural Brain Research*.

[71] Lengenfelder J., Bryant D., Diamond B. J., Kalmar J. H., Moore N. B., DeLuca J. (2006). Processing speed interacts with working memory efficiency in multiple sclerosis. *Archives of Clinical Neuropsychology*.

[72] Salthouse T. A. (2005). Relations between cognitive abilities and measures of executive functioning. *Neuropsychology*.

[73] Sandry J., Simonet D. V., Brandstadter R. (2021). The symbol digit modalities test (SDMT) is sensitive but non-specific in MS: lexical access speed, memory, and information processing speed independently contribute to SDMT performance. *Multiple Sclerosis and Related Disorders*.

[74] McGinley M. P., Goldschmidt C. H., Rae-Grant A. D. (2021). Diagnosis and treatment of multiple sclerosis: a review. *In JAMA - Journal of the American Medical Association*.

[75] Lord S. R., Menz H. B., Tiedemann A. (2003). A physiological profile approach to falls risk assessment and prevention. *In Physical Therapy*.

[76] Hoang P. D., Cameron M. H., Gandevia S. C., Lord S. R. (2014). Neuropsychological, balance, and mobility risk factors for falls in people with multiple sclerosis: a prospective cohort study. *Archives of Physical Medicine and Rehabilitation*.

[77] Prosperini L., Leonardi L., de Carli P., Mannocchi M. L., Pozzilli C. (2010). Visuo-proprioceptive training reduces risk of falls in patients with multiple sclerosis. *Multiple Sclerosis*.

